# The protective effects of melatonin in high glucose environment by alleviating autophagy and apoptosis on primary cortical neurons

**DOI:** 10.1007/s11010-022-04596-w

**Published:** 2022-11-08

**Authors:** Lijiao Xiong, Song Liu, Chaoming Liu, Tianting Guo, Zhihua Huang, Liangdong Li

**Affiliations:** 1 First Affiliated Hospital of Gannan Medical University, Ganzhou, 341000 China; 2 Key Laboratory of Prevention and Treatment of Cardiovascular and Cerebrovascular Diseases of Ministry of Education, Gannan Medical University, Ganzhou, 341000 China; 3 Department of Physiology, Gannan Medical University, Ganzhou, 341000 China; 4 Department of Orthopedics, Guangdong Provincial People’s Hospital Ganzhou Hospital, Ganzhou Municipal Hospital, Ganzhou, 341000 China; 5 Present Address: Xiamen Haicang Biological Science and Technology Development, Xiamen, 361000 China

**Keywords:** Cortical neurons, High glucose environment, Melatonin, Autophagy, Apoptosis

## Abstract

Cognitive dysfunction has been regarded as a complication of diabetes. Melatonin (MLT) shows a neuroprotective effect on various neurological diseases. However, its protective effect on cortical neurons in high glucose environment has not been reported. Our present study aims to observe the protective effect of melatonin on rat cortical neurons and its relationship with autophagy in high glucose environment. The rat primary cortical neurons injury model was induced by high glucose. The CCK-8, flow cytometry, Western blot and immunofluorescence methods were used to examine the cell viability, apoptosis rate and proteins expression. Our results showed that there were no differences in cell viability, apoptosis rate, and protein expression among the control, MLT and mannitol group. The cell viability of the glucose group was significantly lower than that of the control group, and the apoptosis rate of the glucose group was significantly higher than that of the control group. Compared with the glucose group, the glucose + melatonin group showed a significant increase in cell viability and a notable decrease in apoptosis rate. Melatonin concentration of 0.1–1 mmol/L can significantly alleviate the injury of cortical neurons caused by high glucose. Compared with the control group, the glucose group showed a significant reduction of B-cell lymphoma 2 (Bcl-2) protein expression, while remarkable elevations of Bcl2-associated X protein (Bax), cleaved Caspase-3, coiled-coil, myosin-like Bcl2-interacting protein (Beclin-1) and microtubule-associated protein 1 light chain-3B type II (LC3B-II) levels. The neurons pre-administered with melatonin obtained significantly reversed these changes induced by high glucose. The phosphorylation levels of protein kinase B (Akt), mechanistic target of rapamycin kinase (mTOR) and Unc-51 like autophagy activating kinase 1(ULK1) were decreased in the glucose group compared with the control group, whereas significant increase were observed in the glucose + MLT group, compared with the glucose group. These data indicated that melatonin has a neuroprotective effect on cortical neurons under high glucose environment, which may work by activating Akt/mTOR/ULK1 pathway and may be deeply associated with the downregulation of autophagy.

## Introduction

The incidence of diabetes has been increasing in recent years. Traditionally, diabetes complications mainly affect the kidney, retina, peripheral nerve and cardiovascular system. Recently, diabetes cognitive dysfunction has been regarded as a complication of the central nervous system of diabetes. “Diabetes encephalopathy (diabetic encephalopathy, DE)” was first proposed by Reske-Nielsen [1], means the changes in brain structure, function and metabolic state caused by long-term sustained hyperglycemia, which eventually causes nerve behavior and cognitive function defect. It is mainly manifested in the descent of comprehension ability, memory decline, cognitive dysfunction, accompanied by indifference, delayed behavior, and even the development of dementia. Its pathogenesis is related to glucotoxicity, insulin resistance, apoptosis of nerve cells, vascular diseases, oxidative stress, mitochondrial dysfunction, inflammatory response, amyloid deposition and synaptic changes of nerve cells [2]. With high incidence of DE, the search for new effective diagnostic methods and therapeutic measures of DE has important clinical and social application value.

At present, DE has been recognized as an independent type of dementia, and the pathological changes of Tau protein hyperphosphorylation and amyloid protein deposition have also been found in the brains of patients with diabetes. DE and Alzheimer’s disease (AD), Parkinson’s disease (PD), multiple sclerosis and huntingtin’s disease (HD) all belong to neurodegenerative diseases. Abnormal accumulation of proteins in cells formed in the affected brain regions is a common feature of these diseases [3].

When cells are stimulated by the outside environment, they form a membrane-like structure wrapped by a double membrane, transport misfolded proteins or abnormal cell components to lysosomal degradation and maintain cell self-renewal, this process known as macroautophagy. Autophagy is essential for maintaining neuronal homeostasis by clearing damaged mitochondria and phagocytes, such as HD and PD related proteins [4]. Studies have shown that autophagy disorders can lead to degenerative changes in neurons, such as Parkinson’s syndrome and amyotrophic lateral sclerosis [5]. Mitochondrial autophagy plays an important role in apoptosis of nerve cells. In the early stage of nerve cell injury, autophagy can remove harmful substances to protect cells from apoptosis. Excessive activation of autophagy may lead to excessive self-digestion, which in turn triggers the apoptosis process to initiate and promote apoptosis [6]. On account of involved in the pathogenesis of cognitive dysfunction, autophagy regulation has been regarded as a target for the therapy of neurodegenerative disease [4–6].

Studies have shown that the hyperglycemic state of diabetes leads to excessive activation of autophagy, which damages the target organs. For example, in diabetic retinopathy, excessive activation of autophagy by neurons leads to increased apoptosis of retinal nerve cells, while inhibiting their autophagy activity can reduce hyperglycemia toxicity and improve retinal function [7]. Animal experiments showed that the cognitive dysfunction of diabetes also has excessive activation of autophagy, and inhibiting the autophagy activity can improve its cognitive function [8]. Therefore, we speculated that the use of drugs or other methods to inhibit neuronal autophagy could delay the occurrence and development of diabetic cognitive dysfunction.

Melatonin is a hormone synthesized and secreted by pineal gland, which has the physiological functions of regulating sleep, immune regulation, anti-inflammatory and antioxidant. Melatonin has a neuroprotective effect, which can ameliorate brain tissue edema and injury, inhibit neuronal apoptosis, reduce oxidative stress, prevent Aβ aggregation, and so on. It shows protective effects on PD, stroke, AD and etc. in animal model tests [9, 10]. Animal experiments show that melatonin can regulate autophagy and play a neuroprotective role in the rat model of ischemia reperfusion injury [11]. However, whether there is protective effect of melatonin on cortical neurons in high glucose environment has not been reported. Therefore, in our study, the cortical neurons in the high glucose environment were used as the model to simulate the central neuropathy of diabetes mellitus and investigate the protective effect of melatonin and its related mechanisms.

## Materials and methods

### Materials

Female SD rats with pregnancy 18 days weighing 250–280 g were purchased from Hunan Slake Jingda Experimental Animals Limited Liability Company. (license number of animal center: SCXK(Xiang) 2016-0002, grade: SPF). Melatonin (M5250) purchased from Sigma-Aldrich, using Dimethyl sulfoxide (DMSO) solution to dissolve it. Dulbecco’s modified Eagle’s medium (DMEM, C11996600BT), Neurobasal medium (21103-049), B27 supplement (50×) (17504-044), Glutamax™-1(100×) (35050-061) were from Gibco. Fetal bovine serum (FBS), trypsin, penicillin–streptomycin and l-glutamine were from Gibco. Poly-l-lysine (molecular weight 15,000–30,000), glucose, Cell Counting kit-8 (CCK8) (CA1210) was purchased from Solarbio. Apoptosis detection kit (556547) was purchased from BD Pharmingen Company. Bcl-2 (sc-7382) and Bax (sc-20067) antibody were purchased from Santa Cruz Biotechnology (CA, USA). Cleaved caspase-3 (9664S), LC3B (2775S), Beclin-1 (3738S), p-mTOR (5536S), mTOR (2983S), p-Akt (4046S), Akt (9272S), p-ULK1 (14202) and ULK1 (8054) antibody were purchased from Cell Signaling Technology. p-Beclin-1 (ab183335) antibody was purchased from Abcam (Cambridge, UK). β-Tubulin (MA5-11732) antibody was purchased from Invitrogen. Goat anti-rabbit IgG (31460), goat anti-rat IgG (31430), PVDF membrane (88518), protein quantitative reagent (A33972), and enhanced chemiluminescence reagent (34580) were purchased from Thermo Fisher.

### Primary culture of cortical neurons from fetal rats

Experimental protocols for animal studies were approved by the local ethics committee. Primary cortical neurons were isolated and cultured from day E18 Sprague Dawley rat embryos. Anatomical microscope was used to dissect the cortex of rats and put it into Hank’s anatomical solution. Cortex tissues were digested in 0.125% trypsin 18 min at 37 °C water bath, meanwhile oscillation of 2–3 times. DMEM medium containing 10% FBS serum was used to terminate digestion. Centrifuge for 10 min at 1000 rpm/min, and the cells were resuspended with the density of 10^5^/mL and inoculated in the Poly-l-lysine-coated (0.1 g/L) plates, and then cultured in DMEM supplemented with 10% fetal bovine serum, l-glutamine (200 nM), 50 U/mL penicillin and 50 μg/mL streptomycin at 37 °C and 5% CO_2_ incubator. This medium was removed after 4 h and replaced with Neurobasal medium containing 2% B27 supplement. Half the medium was removed every 3 days in culture and replaced with fresh Neurobasal medium with supplements. After 6 days in the supplemented Neurobasal medium, the primary cortical neurons were used for the experiment.

### Cell immunofluorescent staining

Neuronal nuclear protein (NeuN) immunostaining was performed to evaluate the purification rate of cortical neurons. Immunofluorescence of other proteins Bax, Bcl-2, Beclin-1, LC3B and cleaved caspase-3 was performed. The culture medium was removed and the neurons were rinsed for 5 min three times in cold phosphate buffered saline (PBS). Cells were fixed with 4% paraformaldehyde for 30 min, permeabilized in PBS containing 0.2% Triton for 30 min, and blocked with 10% normal goat serum for 1 h. Cells were incubated with mouse anti-Bax, Bcl-2, Beclin-1, LC3B and cleaved caspase-3 primary monoclonal antibody (1:50) for 2 h at 37 °C and washed three times in PBS (5 min per wash), then incubated with goat anti-mouse secondary antibody conjugated to FITC (1:500) for 1 h at 37 °C and washed three times in PBS (5 min per wash). Cells were incubated with DAPI (1:1000) for 5 min at room temperature and examined for fluorescence using a standard fluorescence photomicroscope (DM IRE2, Leica Microsystems, Cambridge, UK).

### Experimental groups

**Fig. 1:**
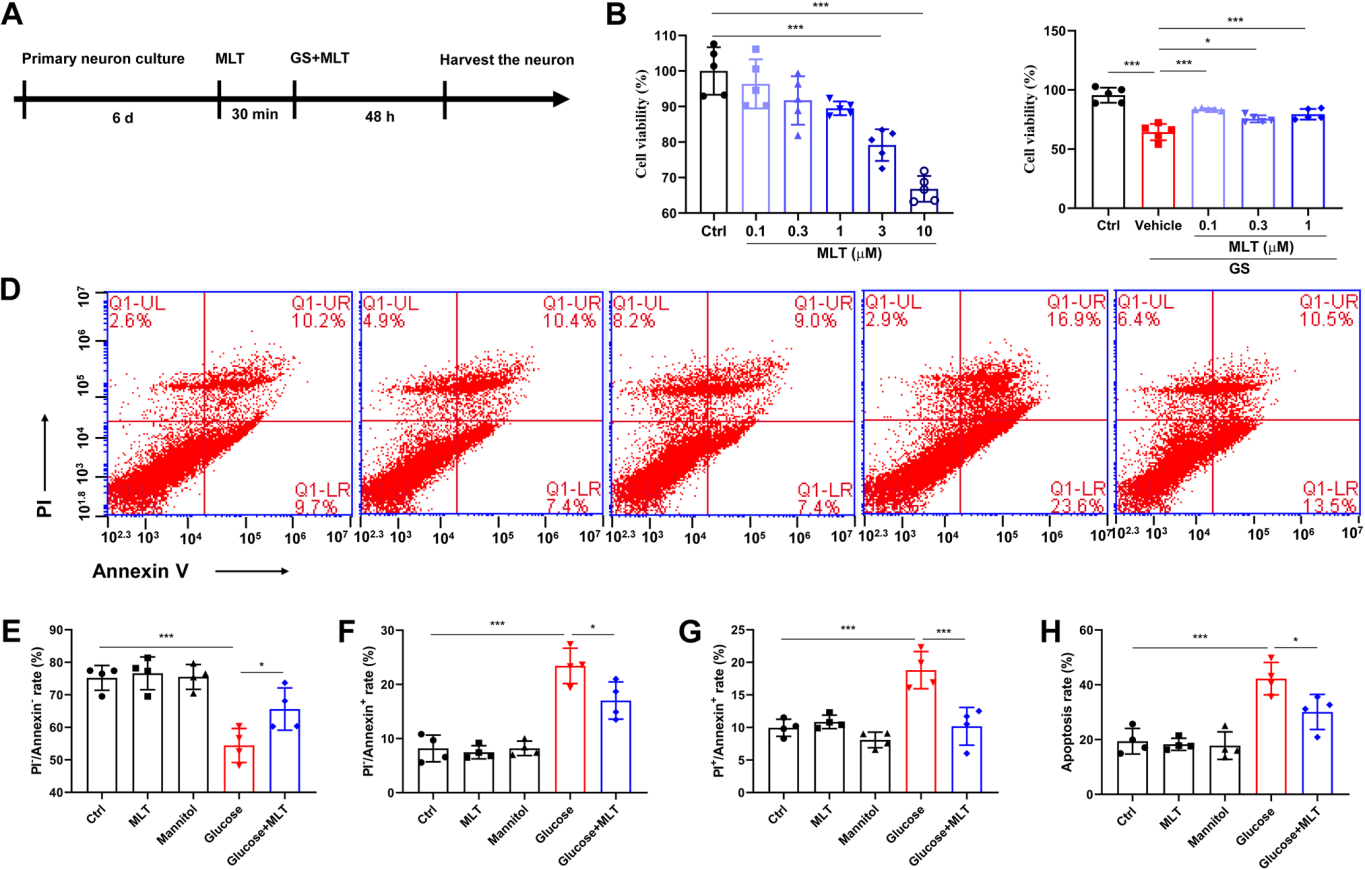
Effects of melatonin on the cell viability and apoptosis of cortical neurons in high glucose environment. **A** Flow chart of the experiment. The primary fetal cortex neurons were cultured and were mature after 6 days. Melatonin was added for pre-administering for half an hour, and then glucose was added for treatment for 48 h. After that, neurons were collected for experimental detection on the 8th day. **B** Cell viability from the six different groups, Control, 0.1 μΜ MLT, 0.3 μΜ MLT, 1 μΜ MLT, 3 μΜ MLT and 10 μΜ MLT, were expressed as mean ± SD. **C** Cell viability from the five different groups, Control, Glucose, Glucose + 0.1 μΜ MLT, Glucose + 0.3 μΜ MLT and Glucose + 1 μΜ MLT, were expressed as mean ± SD. **D** The data of Annexin V/PI double staining collected by flow cytometry were showed as scatter plot. Results demonstrate that MLT protect the primary cultured neurons from subsequent high glucose toxicity injury. **E** The percentage of surviving cells from the different five groups, Control, 0.1 μΜ MLT, Mannitol, Glucose and Glucose + 0.1 μΜ MLT, were showed also as mean ± SD. **F** The percentage of early apoptotic cells. **G** The percentage of late apoptotic cells. **H** The percentage of total apoptotic cells. (*P < 0.05, **P < 0.01, ***P < 0.001)

The cells were divided into control group (Ctrl), melatonin group (MLT), Mannitol group (100 mmol/L), Glucose group (100 mmol/L), and Glucose + MLT group. The primary fetal rat cortical neuron cells were mature after 6 days. Melatonin was added for pretreatment for half an hour, and then glucose or mannitol was added for treatment for 48 h. After that, neurons were collected for experimental detection (Fig. 1A). The concentrations of melatonin in MLT and Glucose + MLT groups were 0.1, 0.3, 1, 3, 10 microns/L in the cell viability assessment. The final concentrations of melatonin in the subsequent experiments were 0.1 μmol/L.

### Cell viability assessment

Cell viability was quantified by CCK-8 assay. The cells were inoculated in 96-well plate with a concentration of 1 × 10^5^/mL, and the treated cells were cultured for 48 h after 7 days. 10 μL CCK-8 was added to each hole, and cultured for 2 h, and the light absorption value was measured at 490 nm on a plate reader, and the neuronal cell viability was calculated as a mean percentage of the control group. All experiments were carried out in quadruplicate.

### Detection of apoptotic cells by Annexin V-FITC/PI flow cytometry

1.5 × 10^6^ cells per pore were plated in six well plates. The cells were cultured for 6 days and then treated with drugs for 48 h. Cells were trypsinized and harvested by centrifugation and then followed the instructions of Annexin V-FITC/PI kit to incubate with Annexin V-FITC and PI for 15 min at room temperature, away from light. Apoptosis rate was examined by flow cytometry in 30 min. The experiment was repeated three times with four duplicate holes in each group.

### Western blot analysis

Primary fetal cortical neurons were processed and cells were collected. The protein concentration in each sample was determined by the bicinchoninic acid (BCA) protein assay and equal amounts of protein per lane were loaded onto the gel. Briefly, samples were electrophoresed on 10% SDS–PAGE gels and transferred to polyvinylidene difluoride membranes. After blocking with Tris hydrochloric acid buffered saline (TBS) containing 0.1% Tween 20 and 5% nonfat milk, the membranes were incubated overnight at 4 °C with a rabbit polyclonal antibody against p-mTOR, mTOR, p-Akt, Akt, p-Beclin-1, Beclin-1, LC3B, Bcl-2, Bax, cleaved caspase-3 and β-tubulin, diluted 1:2000 in 0.05% BSA. They were then washed and incubated with a horseradish peroxidase-conjugated goat-anti-rabbit or goat-anti-mice secondary antibody IgG (1:5000). Protein bands were visualized by chemiluminescence with a Western Chemiluminescent HRP Substrate kit (Immobilon) and the band images analyzed with Image J software (National Institutes of Health, Bethesda, MD, USA). β-Actin was used as a control for the protein loading. Each experiment was repeated at least three times.

### Statistical analysis

All statistical analyses were carried out with GraphPad Prism 5.0 software. Data are expressed as mean ± SD and analyzed by one-way ANOVA followed by post hoc Student–Newman–Keuls test to determine the influence of different groups. *P* < 0.05 was considered statistically significant.

## Results

### Effect of melatonin on the cell viability and apoptosis of cortical neurons in high glucose environment

When melatonin concentration was 0.1, 0.3 and 1 µmol/L, the survival rate of cortical neurons was not statistically different from that of the control group (*P* > 0.05). When melatonin concentration was 3 and 10 µmol/L, the cell survival rate was significantly lower than that of the control group, which indicating that 3 and 10 µmol/L MLT had toxic effects on primary cultured cortical neurons (Fig. 1B). The survival rate of glucose group was significantly lower than that of the control group (*P* < 0.01). The results of cell viability assay revealed that treatment with the MLT markedly protected the cortical neurons from injury caused by high glucose, and improved the cell survival rate (Fig. 1C). There was no statistical difference in apoptosis rate between the control group, melatonin group and mannitol group (*P* > 0.05). The apoptosis rate in the Glucose group was significantly higher than that in the control group (*P* < 0.05), and significantly lower in the Glucose + MLT group than that in the Glucose group (*P* < 0.05) (Fig. 1D–H). This indicated that melatonin concentration of 0.1–1 µM can alleviate the damage of cortical neurons induced by high glucose, while melatonin concentration of 3 and 10 µM MLT can have toxic effects on the original cultured cortical neurons.

### Effects of melatonin on the apoptosis-related proteins expression on cortical neurons in high-glucose environment

**Fig. 2:**
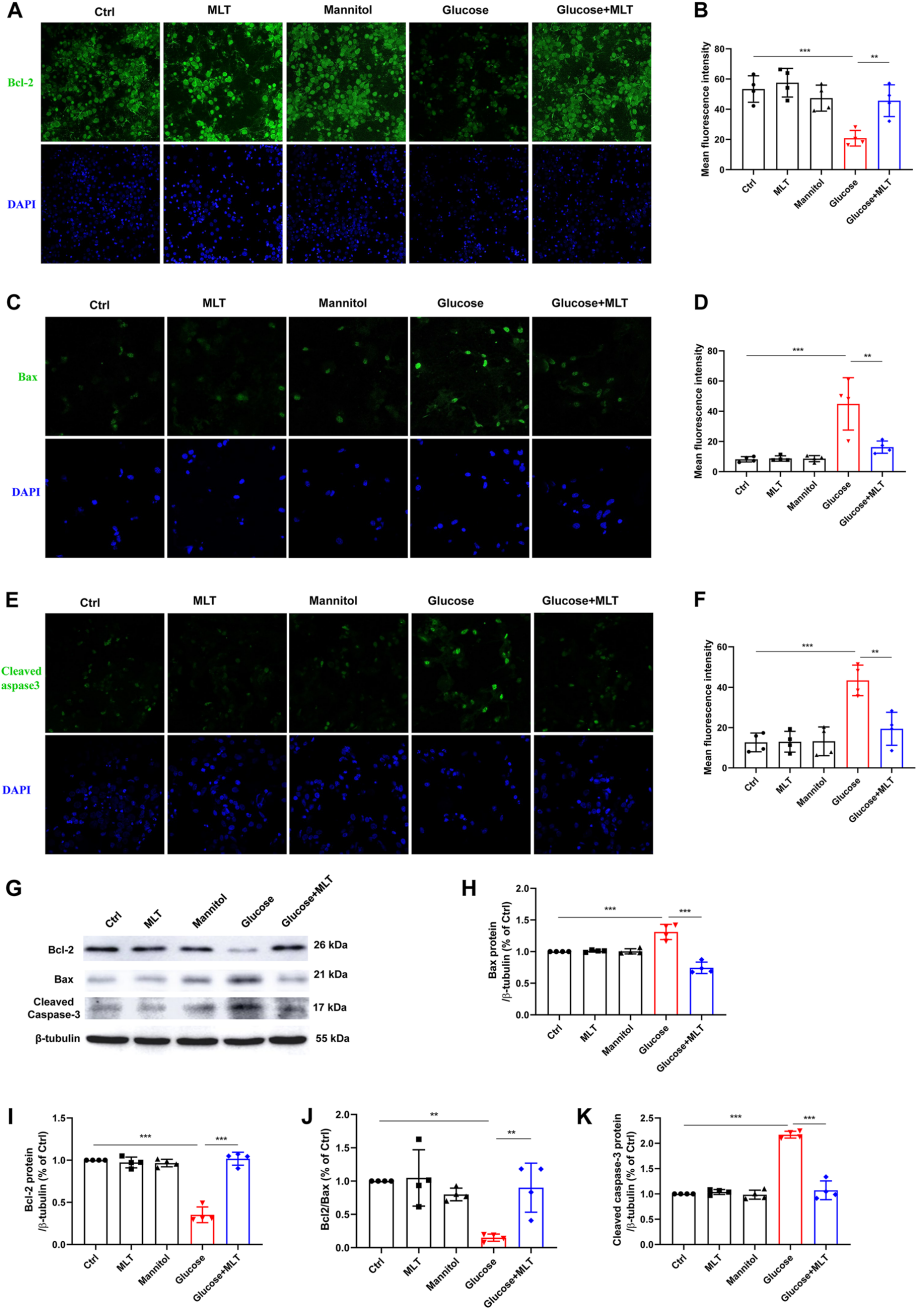
Effects of melatonin on the apoptosis-related proteins expression on cortical neurons in high-glucose environment. The neuronal cells were cultured in 60 mm dish (4.5 × 10^6^/well) for Western blot assay, and were cultured on glass sheet in 24 well plates (1 × 10^5^/well) for immunofluorescence test. **A**–**F** Bcl-2, Bax and cleaved caspase-3 localization and quantitative statistical charts were showed as fluorescent photo. **G**–**K** Bcl-2, Bax and cleaved caspase-3 protein level from the five different groups, control, 0.1 μΜ MLT, Mannitol, Glucose and Glucose + 0.1 μΜ MLT, were expressed as mean ± SD. Results demonstrate that MLT decreases Bax and cleaved caspase-3 level, and increases Bcl-2 proteins expression and Bcl-2/Bax ratio in primary cortical neurons injured by high glucose. (*P < 0.05, **P < 0.01, ***P < 0.001)

There was no significant difference in protein expression of Bcl-2, Bax and cleaved caspase-3 among the control, melatonin and mannitol group by Western blot and immunofluorescence (*P* > 0.05) (Fig. 2A–K). Western blot and immunofluorescence both showed that the expression of Bcl-2 was significantly decreased while the expression of Bax and cleaved caspase-3 was significantly increased in the glucose group compared with the control group, showing a statistically significant difference (*P* < 0.05). Compared with the glucose group, glucose + MLT group obtained a significantly increase in Bcl-2 protein expression, while significantly decreases in Bax and cleaved caspase-3 expression with statistical significant difference (*P* < 0.05) (Fig. 2A–K). These results indicate that MLT regulate the expression of apoptosis relation protein and inhibit cell apoptosis induced by high glucose stimulation.

### Effects of melatonin on the autophagy related proteins expression on cortical neurons in high-glucose environment

**Fig. 3:**
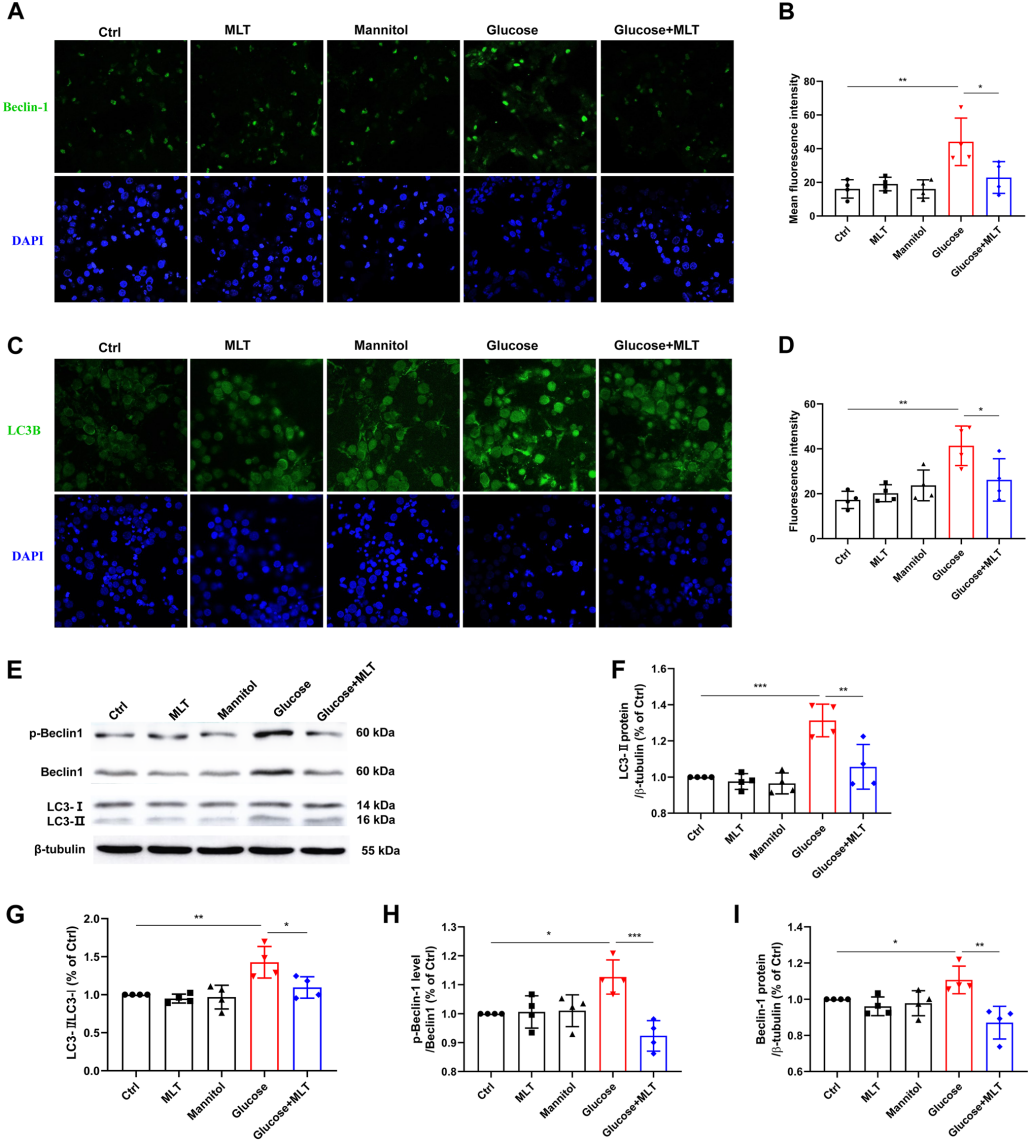
Effects of melatonin on the autophagy related proteins expression on cortical neurons in high-glucose environment. **A**–**D** Beclin-1 and LC3B-II localization and quantitative statistical charts were showed as fluorescent photo. **E**–**I** Beclin-1, p-Beclin-1 and LC3B protein level from the five different groups, control, 0.1 μΜ MLT, Mannitol, Glucose and Glucose + 0.1 μΜ MLT, were expressed as mean ± SD. Results demonstrate that MLT decreases Beclin-1, p-Beclin-1 and LC3B-II proteins expression and LC3B-II/ LC3B-II ratio in primary cortical neurons injured by high glucose. (*P < 0.05, **P < 0.01, ***P < 0.001)

Western blot and immunofluorescence both showed that there was no statistical difference on the expression of autophagy related proteins Beclin-1, LC3B-II and LC3B-II/LC3B-I among the control, melatonin, mannitol group (Fig. 3A–I). Immunofluorescence results showed that Beclin-1 and LC3B expression in the glucose group were significantly higher than the control group. Beclin-1 and LC3B expression were significantly lower in the glucose + MLT group than in the glucose group, and the difference was statistically significant (Fig. 3A–D). Western blot results show that the expression of p-Beclin-1, LC3B-II and LC3B-II/LC3B-I were significantly increased in the glucose group compared with control group, with a statistically significant difference (*P* < 0.01). Compared with the glucose group, glucose + MLT showed significant reductions in the expression of p-Beclin-1, LC3B-II and LC3B-II/LC3B-I (*P* < 0.01) (Fig. 3E–I). The results of Western blot and immunofluorescence were consistent.

### Effects of melatonin on the phosphorylation of Akt/mTOR/ULK1 protein on cortical neurons in high-glucose environment

**Fig. 4:**
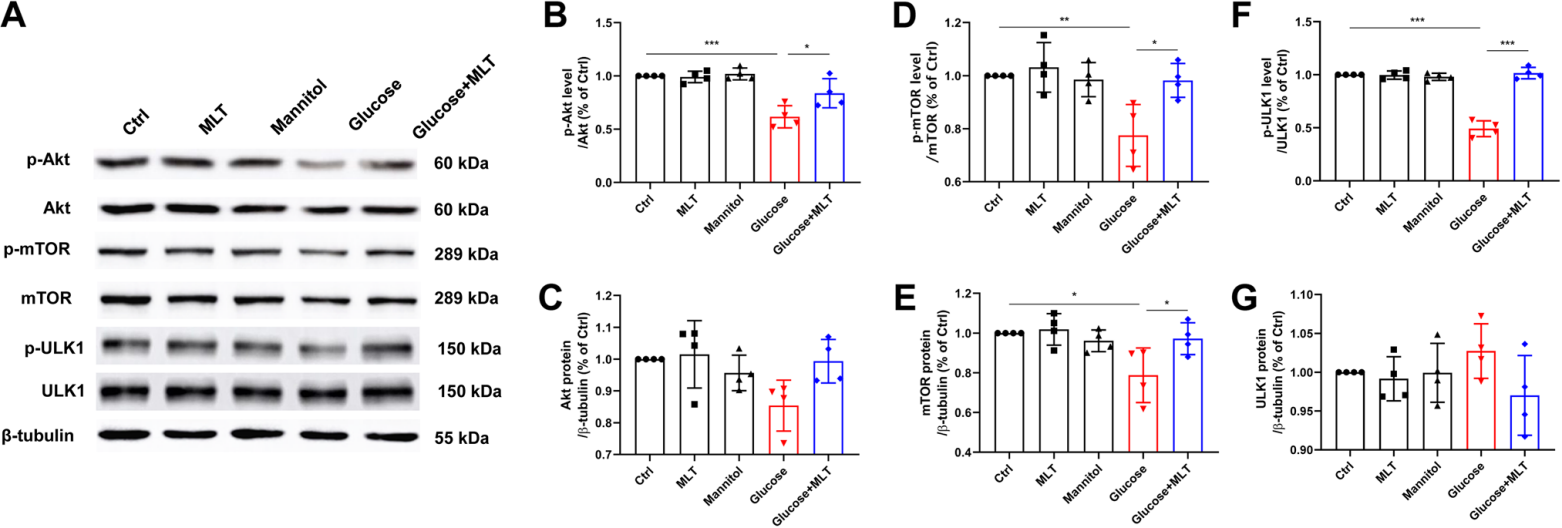
Effects of melatonin on the phosphorylation of Akt/mTOR/ULK1 protein on cortical neurons in high-glucose environment by Western blot. **A**–**G** p-Akt, Akt, p-mTOR, mTOR, p-ULK1 and ULK1 protein level from the different groups, control, 0.1 μΜ MLT, Mannitol, Glucose and Glucose + 0.1 μΜ MLT, were expressed as mean ± SD. Results demonstrate that MLT increases p-Akt, p-mTOR, mTOR and p-ULK1 proteins expression in primary cortical neurons injured by high glucose. (*P < 0.05, **P < 0.01, ***P < 0.001)

The phosphorylation and total of Akt, mTOR and ULK1 protein in each group was determined by Western blot, and there was no statistically significant difference among the control, melatonin and mannitol group (*P* > 0.05) (Fig. 4A–G). p-Akt, p-mTOR, mTOR and p-ULK1 and ULK1 (were significantly decreased in the glucose group compared with the control group (*P* < 0.05), and were significantly increased in the glucose + MLT group compared with the glucose group (*P* < 0.05) (Fig. 4A–G). It suggested that Akt/mTOR/ULK1 activation was significantly inhibited in the glucose group, and melatonin could increase the phosphorylation level of Akt/mTOR/ULK1 in cortical neurons in the high glucose environment.

## Discussion

The pathogenesis of diabetic central neuropathy is not completely clear. The high fatty acids, high blood glucose and abnormal increase of glycation end products caused by hyperglycemia induce oxidative stress. Excess produced or insufficient eliminated ROS will directly cause biological membrane lipid peroxidation, protein and enzyme degeneration, mitochondrial dysfunction, which lead to neuronal cell damage, scabbard film, axon swelling degeneration and even broke off, neurons chromatin dissolved, cytoplasm vacuoles degeneration necrosis, mitochondrial dysfunction, nuclear pyknosis and nerve cells apoptosis [3]. Abnormal changes in brain electrophysiology, imaging and pathology caused by diabetic metabolic factors such as glucose and lipid metabolism disorder, insulin resistance, vascular disease and obesity are closely related to the occurrence and development of DE.

Melatonin is a strong antioxidant, with small molecular volume and amphiphilic nature, which is very easy to enter the cells, especially the mitochondria, and participate in mitochondrial energy metabolism to reduce mitochondrial damage caused by oxidative stress [12–14]. Relevant studies have shown that melatonin can directly interact with electrons or induce the production of antioxidant enzymes to reduce the damage of oxidative respiratory chain, enhance mitochondrial function and reduce oxidative stress even reduce cell apoptosis [12]. In addition, melatonin can help restore the function of islet B cells, improve insulin sensitivity and glucose tolerance, reduce hyperinsulinemia, and reduce insulin resistance. Melatonin may be a potential diabetes treatment drug.

In the hippocampus and cortex of diabetic rats, lipid peroxides increased, and rats showed obvious cognitive impairment. However, these manifestations were significantly reduced or even disappeared after the treatment with melatonin or antioxidants vitamin E [15, 16]. Melatonin can also alleviate peripheral neuralgia caused by diabetes and improve diabetic neuropathy [17]. In addition, melatonin has neuroprotective effects. Patients suffered from AD showed a reduction in secretion of melatonin, which slowed down the synthesis of the protein of Aβ protein and delay the progression of this disease [18]. Activating the receptor of melatonin presented a protective effect on spinal cord injury [19]. In addition, melatonin showed satisfactory neuroprotection and antioxidation effects on cultured primary cortical neurons deprived of oxygen and sugar, and animal brain injury induced by cerebral ischemic stroke [20]. In this study, we found that melatonin could improve the survival rate of cortical neurons and reduce the apoptosis rate of neurons in fetal rats under high-glucose environment, indicating that melatonin could protect neurons in cortex of fetal rats under high glucose environment.

The cerebral cortex is an important anatomical basis for learning and memory and it is vulnerable to various adverse factors. The thickness and volume of cerebral cortex were significantly reduced in diabetic patients, which is the structural basis for learning and memory impairment in diabetic patients [21]. High glucose environment can cause apoptosis of cortex and hippocampal neurons [22, 23]. Bax and Bcl-2 family are very important apoptotic regulatory genes. Bax is apoptosis promoting gene, and Bcl-2 is apoptosis inhibiting gene. In addition, Bcl-2 protein family can not only regulate cell apoptosis but also affect other cell processes, such as cell cycle, calcium signaling, glucose stability, autophagy and etc., which play an important role in cell survival and death [24]. Caspase-3 is the main executor of apoptosis, and cleaved caspase-3 can cause downstream apoptosis cascade reaction. In this study, we found that the high glucose environment can lead to apoptosis of cortical neurons, and regulated the expression of Bax, cleaved caspase-3 and Bcl-2, and the hypertonic state of mannitol had no effect on neuronal apoptosis and protein levels, suggesting that it is high glucose rather than hyperosmosis directly leads to apoptosis and the changes of relevant protein expression in neurons, Melatonin can reverse the change of Bax, cleaved caspase-3 and bcl-2 proteins in cortical neurons induced by high-glucose environment and reduce neuron apoptosis.

Autophagy is an important degradation pathway for phagocytosis of aging proteins or organelles in the cytoplasm, which is degraded by autophagy lysosome to complete the metabolic needs of the body and the renewal of aging organelles [25]. Different from other cells, neurons don’t have the function of re-division, and can’t dilute harmful substances in cells by way of division. Therefore, the autophagic lysosome degradation pathway is particularly important in neurons [4]. At present, studies have found that the abnormal autophagy function plays an important role in the occurrence and development of nervous system diseases, such as PD and HD. The regulation of autophagy activity can delay the progress of the disease through devouring damaged mitochondria, reducing oxidative stress and inhibiting apoptosis [5, 6]. The excessive activation of autophagy can promote apoptosis. In addition, autophagy and apoptosis can exist simultaneously and interact with each other [26]. Inhibition of autophagy activity in cerebral ischemia reperfusion rats can improve their neural function [27]. At present, there are few studies on autophagy in diabetic encephalopathy, and changes in autophagy activity of cells may be one of the important mechanisms of diabetic encephalopathy [8, 28, 29]. Many studies have shown that the neuron apoptosis and cognitive decline in diabetic rats are related to the excessive activation of autophagy, and inhibition of autophagy activity can improve the cognitive function of diabetic mice [8, 28, 29]. Diabetic retinopathy and diabetic hearing impairment were associated with elevated levels of autophagy in diabetic nerve cells [7, 30]. This study showed that the expression of Beclin-1 and LC3B-II, two landmark molecules of autophagy, could be increased in high glucose environment, suggesting that high glucose environment could induce autophagy. Melatonin can decrease Beclin-1 and LC3B-II/LC3B-I ratio in high glucose environment, and reduce the apoptosis rate of cortical neurons. It suggests that melatonin can protect cortical neurons by downregulating autophagy activity. This study is similar to the results of other studies [8, 28, 29]. However, some studies showed that the autophagy activity of neurons in diabetic neuropathy and diabetic rats with microvascular disease decreases, and the activation of autophagy can reduce the damage of neurons [31–33]. We believe that the differences in these findings are related to the degree of autophagy activation. Moderate autophagy activation has neuroprotective effects, but excessive autophagy activation can induce programmed cell death.

Akt/mTOR/ULK1 signaling pathway is an important mechanism for the negative regulation of autophagy. Bcl-2 inhibits autophagy initiation by binding to Beclin-1 in the structural domain of BH3 protein, and mTOR is required to participate in the binding of Beclin1 to BH3 protein. Activation of mTOR phosphorylates ULK1 at Ser757, a crucial inducer of the initiation of autophagy, arrests the interaction between ULK1 and AMPK through ULK1-ATG13-FIP200-ATG101 complex, directly inhibits autophagic initiation, and regulates autophagic related protein phosphorylation and thus blocks autophagy [34–36]. Studies found that diabetic cognitive dysfunction is related to mTOR signaling pathway_,_ and activation of PI3K/Akt/mTOR signaling pathway can improve nerve defects in diabetic cerebral ischemia reperfusion rats and hyperglycemia induced neurotoxicity of PC-12 cells [23, 37]. In addition, activation of Akt/mTOR signaling pathway can promote the growth of posterior axons and improve the functional recovery after stroke [38]. It also found that melatonin can regulate AMPK/mTOR signaling pathway and play a protective role in myocardial ischemia reperfusion [14], reduce the neuron apoptosis induced by focal cerebral ischemia in mice via activating the PI3K/Akt signaling pathway [39], and alleviate nerve defects caused by middle cerebral artery occlusion in adult male rats through activating Akt/mTOR signaling pathway [40]. Therefore, we believe that melatonin can regulate Akt/mTOR signaling pathway, which has also been demonstrated in this study. In this study, we found that high glucose environment inhibited the Akt/mTOR/ULK1 signaling pathway of cortical neurons, reduced the expression of Bcl-2, and over-activated the autophagy level of cortical neurons. Melatonin can activate Akt/mTOR/ULK1 pathway, upregulate the expression of Bcl-2, inhibit the over-activation of autophagy and show neuroprotective effects.

To sum up, melatonin can reduce the apoptosis of cortical neurons under the high-sugar environment by activating Akt/mTOR/ULK1 pathway and being related with the down-regulation of autophagy, which has a neuroprotective effect.

## Data Availability

The data that support the findings of this study are available from the corresponding author upon reasonable request.

## References

[1] Reske-Nielsen E, Lundbæk K, Rafaelsen OJ (1966). Pathological changes in the central and peripheral nervous system of young long-term diabetics: I. Diabetic encephalopathy. Diabetologia.

[2] Vieira LL, de Lima Soares RG, Da Silva Felipe SM, de Moura FC, de Castro Brito GA, Pacheco C, Soares PM (2017). Physiological targets for the treatment of diabetic encephalopathy. Cent Nerv Syst Agents Med Chem.

[3] Zilliox LA, Chadrasekaran K, Kwan JY, Russell JW (2016). Diabetes and cognitive impairment. Curr Diab Rep.

[4] Nixon RA (2013). The role of autophagy in neurodegenerative disease. Nat Med.

[5] Jiang PD, Mizushima N (2014). Autophagy and human diseases. Cell Res.

[6] Ghavami S, Shojaeid S, Yeganeh B, Ande SR, Jangamreddy JR, Mehrpour M, Christoffersson J, Chaabane W, Moghadam AR, Kashani HH, Hashemi M, Owji AA, Los MJ (2014). Autophagy and apoptosis dysfunction in neurodegenerative disorders. Prog Neurobiol.

[7] Wang W, Wang Q, Wan D, Sun Y, Wang L, Chen H, Liu C, Petersen RB, Li J, Xue W, Zheng L, Huang K (2017). Histone HIST1H1C/H1.2 regulates autophagy in the development of diabetic retinopathy. Autophage.

[8] Ma L, Lv Y, Huo K, Liu J, Shang S, Fei Y, Li Y, Zhao B, Wei M, Deng Y, Qu Q (2017). Autophagy-lysosome dysfunction is involved in A beta deposition in STZ-induced diabetic rats. Behav Brain Res.

[9] Kim HA, Lee KH, Lee BH (2014). Neuroprotective effect of melatonin against kainic acid-induced oxidative injury in hippocampal slice culture of rats. Int J Mol Sci.

[10] Dehdashtian E, Mehrzadi S, Yousefi B, Hosseinzadeh A, Reiter RJ, Safa M, Ghaznavi H, Naseripour M (2018). Diabetic retinopathy pathogenesis and the ameliorating effects of melatonin; involvement of autophagy, inflammation and oxidative stress. Life Sci.

[11] Roohbakhsh A, Shamsizadeh A, Hayes AW, Reiter RJ, Karimi G (2018). Melatonin as an endogenous regulator of diseases: the role of autophagy. Pharmacol Res.

[12] Ganie SA, Dar TA, Bhat AH, Dar KB, Anees S, Zargar MA, Masood A (2016). Melatonin: a potential anti-oxidant therapeutic agent for mitochondrial dysfunctions and related disorders. Rejuvenation Res.

[13] Paradies G, Paradies V, Ruggiero FM, Petrosillo G (2015). Protective role of melatonin in mitochondrial dysfunction and related disorders. Arch Toxicol.

[14] Chen WR, Liu HB, Chen YD, Sha Y, Ma Q, Zhu PJ, Mu Y (2018). Melatonin attenuates myocardial ischemia/reperfusion injury by inhibiting autophagy via an AMPK/mTOR signaling pathway. Cell Physiol Biochem.

[15] Tuzcu M, Baydas G (2006). Effect of melatonin and vitamin E on diabetes-induced learning and memory impairment in rats. Eur J Pharmacol.

[16] Olajide OJ, Asogwa NT, Moses BO, Oyegbola CB (2017). Multidirectional inhibition of cortico-hippocampal neurodegeneration by kolaviron treatment in rats. Metab Brain Dis.

[17] Negi G, Kumar A, Kaundal RK, Gulati A, Sharma SS (2010). Functional and biochemical evidence indicating beneficial effect of Melatonin and Nicotinamide alone and in combination in experimental diabetic neuropathy. Neuropharmacology.

[18] Buendia I, Parada E, Navarro E, Leon R, Negredo P, Egea J, Lopez MG (2016). Subthreshold concentrations of melatonin and galantamine improves pathological AD-Hallmarks in hippocampal organotypic cultures. Mol Neurobiol.

[19] Gao Y, Bai C, Zheng D, Li C, Zhang W, Li M, Guan W, Ma Y (2016). Combination of melatonin and Wnt-4 promotes neural cell differentiation in bovine amniotic epithelial cells and recovery from spinal cord injury. J Pineal Res.

[20] Parada E, Buendia I, Leon R, Negredo P, Romero A, Cuadrado A, Lopez MG, Egea J (2014). Neuroprotective effect of melatonin against ischemia is partially mediated by alpha-7 nicotinic receptor modulation and HO-1 overexpression. J Pineal Res.

[21] Geijselaers SL, Sep SJ, Stehouwer CD, Biessels GJ (2015). Glucose regulation, cognition, and brain MRI in type 2 diabetes: a systematic review. Lancet Diabetes Endocrinol.

[22] Qi WW, Zhong LY, Li XR, Li G, Liu ZX, Hu JF, Chen NH (2012). Hyperglycemia induces the variations of 11beta-hydroxysteroid dehydrogenase type 1 and peroxisome proliferator-activated receptor-gamma expression in hippocampus and hypothalamus of diabetic rats. Exp Diabetes Res.

[23] Wu J, Zhou SL, Pi LH, Shi XJ, Ma LR, Chen Z, Qu ML, Li X, Nie SD, Liao DF, Pei JJ, Wang S (2017). High glucose induces formation of tau hyperphosphorylation via Cav-1-mTOR pathway: a potential molecular mechanism for diabetes-induced cognitive dysfunction. Oncotarget.

[24] Strappazzon F, Vietri-Rudan M, Campello S, Nazio F, Florenzano F, Fimia GM, Piacentini M, Levine B, Cecconi F (2011). Mitochondrial BCL-2 inhibits AMBRA1-induced autophagy. EMBO J.

[25] Tooze SA, Codogno P (2011). Compartmentalized regulation of autophagy regulators: fine-tuning AMBRA1 by Bcl-2. EMBO J.

[26] Yang M, Yang XM, Yin DH, Tang QL, Wang L, Huang C, Li P, Li SS (2018). Beclin1 enhances cisplatin-induced apoptosis via Bcl-2-modulated autophagy in laryngeal carcinoma cells Hep-2. Neoplasma.

[27] Wang J, Mei Z, Fu Y, Yang S, Zhang S, Huang W, Xiong L, Zhou H, Tao W, Feng Z (2018). Puerarin protects rat brain against ischemia/reperfusion injury by suppressing autophagy via the AMPK-mTOR-ULK1 signaling pathway. Neural Regen Res.

[28] Kong F, Ma L, Guo J, Xu L, Li Y, Qu S (2018). Endoplasmic reticulum stress/autophagy pathway is involved in diabetes-induced neuronal apoptosis and cognitive decline in mice. Clin Sci.

[29] Li X, Hue Q, Chen L, Liu S, Su S, Tao H, Zhang L, Sun T, He L (2017). HSPB8 promotes the fusion of autophagosome and lysosome during autophagy in diabetic neurons. Int J Med Sci.

[30] Huang X, Zhang W, Yuan Y, Liu X, Yang C, Li Q, Zeng J, Zhou K, Liang Y (2017). Implication of autophagy and apoptosis in spiral ganglion cells and cochlear nucleus nuerons in diabetes-induced hearing impairment in rats. Biomed Res India.

[31] Liu S, Chen L, Li X, Hu Q, He L (2018). *Lycium barbarum* polysaccharide protects diabetic peripheral neuropathy by enhancing autophagy via mTOR/p70S6K inhibition in Streptozotocin-induced diabetic rats. J Chem Neuroanat.

[32] Guan Z, Tao Y, Zhang X, Guo Q, Liu Y, Zhang Y, Wang Y, Ji G, Wu G, Wang N, Yang H, Yu Z, Guo J, Zhou H (2017). G-CSF and cognitive dysfunction in elderly diabetic mice with cerebral small vessel disease: preventive intervention effects and underlying mechanisms. CNS Neurosci Ther.

[33] Areti A, Komirishetty P, Akuthota M, Malik RA, Kumar A (2017). Melatonin prevents mitochondrial dysfunction and promotes neuroprotection by inducing autophagy during oxaliplatin-evoked peripheral neuropathy. J Pineal Res.

[34] Zhang F, Wang J, Chu J, Yang C, Xiao H, Zhao C, Sun Z, Gao X, Chen G, Han Z, Zou W, Liu T (2015). MicroRNA-146a induced by hypoxia promotes chondrocyte autophagy through Bcl-2. Cell Physiol Biochem.

[35] Ryskalin L, Limanaqi F, Frati A, Busceti CL, Fornai F (2018). mTOR-related brain dysfunctions in neuropsychiatric disorders. Int J Mol Sci.

[36] Xiang H, Zhang J, Lin C, Zhang L, Liu B, Ouyang L (2020). Targeting autophagy-related protein kinases for potential therapeutic purpose. Acta Pharm Sin B.

[37] Pan Y, Wang N, Xia P, Wang E, Guo Q, Ye Z (2018). Inhibition of Rac1 ameliorates neuronal oxidative stress damage via reducing Bcl-2/Rac1 complex formation in mitochondria through PI3K/Akt/mTOR pathway. Exp Neurol.

[38] Mao L, Jia J, Zhou X, Xiao Y, Wang Y, Mao X, Zhen X, Guan Y, Alkayed NJ, Cheng J (2013). Delayed administration of a PTEN inhibitor BPV improves functional recovery after experimental stroke. Neuroscience.

[39] Kilic U, Caglayan AB, Beker MC, Gunal MY, Caglayan B, Yalcin E, Kelestemur T, Gundogdu RZ, Yulug B, Yilmaz B, Kerman BE, Kilic E (2017). Particular phosphorylation of PI3K/Akt on Thr308 via PDK-1 and PTEN mediates melatonin’s neuroprotective activity after focal cerebral ischemia in mice. Redox Biol.

[40] Koh P (2008). Melatonin prevents ischemic brain injury through activation of the mTOR/p70S6 kinase signaling pathway. Neurosci Lett.

